# Influence of Estrogenic Metabolic Pathway Genes Polymorphisms on Postmenopausal Breast Cancer Risk

**DOI:** 10.3390/ph14020094

**Published:** 2021-01-27

**Authors:** Micaela Almeida, Mafalda Soares, José Fonseca-Moutinho, Ana Cristina Ramalhinho, Luiza Breitenfeld

**Affiliations:** 1 Health Sciences Research Centre (CICS), Faculty of Health Sciences, University of Beira Interior (UBI), Avenida Infante D. Henrique, 6200-506 Covilhã, Portugal; micaelacpalmeida@gmail.com (M.A.); mafalda.n.soares@gmail.com (M.S.); jafmoutinho@fcsaude.ubi.pt (J.F.-M.); cramalhinho@fcsaude.ubi.pt (A.C.R.); 2 Academic Hospital of Cova da Beira (CHUCB), Quinta do Alvito, 6200-251 Covilhã, Portugal

**Keywords:** breast cancer, *GSTM1*, *GSTT1*, *CYP1B1*, *MTHFR*

## Abstract

Estrogen metabolism plays an important role in tumor initiation and development. Lifetime exposure to high estrogens levels and deregulation of enzymes involved in estrogen biosynthetic and metabolic pathway are considered risk factors for breast cancer. The present study aimed to evaluate the impact of mutations acquisition during the lifetime in low penetrance genes that codify enzymes responsible for estrogen detoxification. Genotype analysis of *GSTM1* and *GSTT1* null polymorphisms, *CYP1B1* Val432Leu and *MTHFR* C677T polymorphisms was performed in 157 samples of women with hormone-dependent breast cancer and correlated with the age at diagnosis. The majority of patients with *GSTT1* null genotype and with both *GSTM1* and *GSTT1* null genotypes were 50 years old or more at the diagnosis (*p*-value = 0.021 and 0.018, respectively). Older women with *GSTM1* null genotype were also carriers of the *CYP1B1*Val allele (*p*-value = 0.012). As well, *GSTT1* null and *CYP1B1*Val genotypes were correlated with diagnosis at later ages (*p*-value = 0.022). Similar results were found associating *MTHFR* C677T and *GSTT1* null polymorphism (*p*-value = 0.034). Our results suggest that estrogen metabolic pathway polymorphisms constitute a factor to be considered simultaneously with models for breast cancer risk assessment.

## 1. Introduction

Breast cancer is the most common cancer in women, counting 2.1 million cases diagnosed in 2018 [1]. Breast cancer risk has long been associated with reproductive and hormonal history, lifestyle and hereditary [2]. Mainly, these risk factors are related to exposure to high levels of endogenous or exogenous estrogens and to mutations inherited or acquired during lifetime [3].

Estrogens metabolism plays an important role in tumor initiation and development, estrogen being considered a carcinogen [4]. This fact is not only due to the exposure to high levels of estrogen and to estrogen receptor (ER) status, to which estrogen binds to exerts its actions, but also to the possible deregulation of the enzymes involved in the estrogen biosynthetic and metabolic pathway, such as Cytochrome P450, family 1, subfamily B, polypeptide 1 (CYP1B1), glutathione S-transferases (GSTs) and 5,10-methylenetetrahydrofolate reductase (MTHFR) [5,6,7,8].

**Figure 1:**
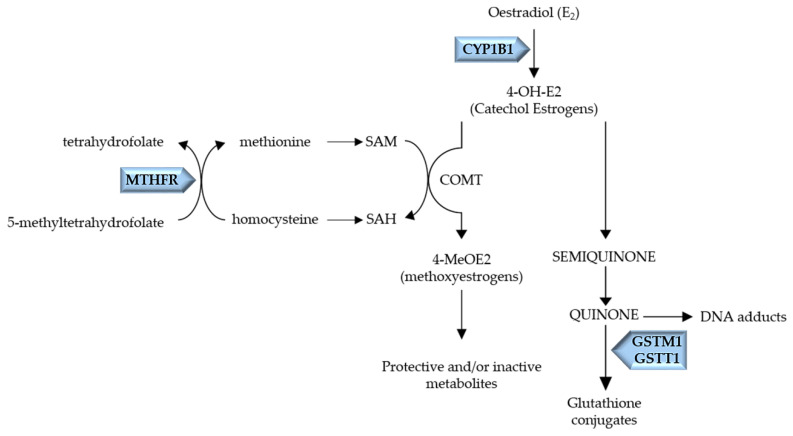
Schematic representation of the metabolic pathway of Oestradiol. CYP1B1, a Phase I enzyme, codified by the *CYP1B1* gene, leads to 4-OH-E2 production. The Phase II enzymes, COMT, codified by the *COMT* gene, and GSTM1/GSTT1, codified by *GSTM1* and *GSTT1* genes, respectively, inactivate the estrogen catechol, semiquinone and quinone, diminishing DNA adducts formation. MTHFR, an enzyme of the folate metabolism, catalyzes 5,10-methylenetetrahydrofolate to 5-methyltetrahydrofolate, which allows the remethylation of homocysteine to methionine, a precursor of S-adenosylmethionine (SAM). SAM is the methyl donor for COMT catalyzed reactions, allowing the inactivation of catechol estrogens.

CYP1B1, codified by the *CYP1B1* gene, located on chromosome 2p21–p22, is a Phase I enzyme, responsible for generating 4-hydroxyestradiol (4-OH-E2), a catechol estrogen metabolite (Figure 1) [9].

An important polymorphism in *CYP1B1* is Val432Leu polymorphism, in exon 3 (rs1056836; location chromosome 2:38071060) [10]. This polymorphism leads to an amino acid substitution of Leucine to Valine, Val432 allele was found to increase the 4-hydroxilase activity of CYP1B1 [11]. A lack or low level of Phase II detoxifying enzymes, such as catechol-O-methyltransferase (COMT) and GSTs, might lead to quinones accumulation, resulting in DNA adducts formation and tumor initiation [3]. Also, MTHFR is involved in the conjugation and inactivation of catechol estrogens by COMT. MTHFR is the key enzyme of folate metabolism, catalyzing 5,10-methylenetetrahydrofolate to 5-methyltetrahydrofolate, which allows the remethylation of homocysteine to methionine, a precursor of S-adenosylmethionine (SAM) [12]. In turn, SAM is the methyl donor for COMT catalyzed reactions, which allow inactivation of catechol estrogens [12]. In this regard, MTHFR activity alterations will affect indirectly the inactivation of catechol estrogens by COMT. MTHFR is codified by the *MTHFR* gene, located on chromosome 1p36.3 [13]. One of the well-studied polymorphisms of *MTHFR* is the polymorphism C677T (rs1801133, location chromosome 1:11796321), which leads to a substitution of alanine with valine, being correlated with lower activity of MTHFR [13,14]. Thus, this polymorphism possibly affects COMT catalyzed reactions, compromising the catalyzation of 4-OH-E2 to 4-methoxyestrogens (4-MeOE2) [12].

The GSTs, a superfamily of Phase II enzymes, are responsible for metabolic detoxification of estrogen, playing a key role in the catalysis of glutathione (GSH) conjugation with catechol estrogen quinones, which are rapidly excreted by the cell [3]. There are seven classes of cytosolic GSTs: alpha (α), mu (μ), kappa (κ), pi (π), theta (θ), omega (Ω) and zeta (ζ) [15]. Among the polymorphisms studied in GSTs, Glutathione S-transferase Theta1 (*GSTT1*) and Glutathione S-transferase Mu1 (*GSTM1*) null polymorphisms, the homozygous genotype of which implies the total absence of the enzyme, has previously been related to breast cancer risk by our research team [16,17]. GSTM1 and GSTT1 enzymes are codified by the *GSTM1* gene located on chromosome 1p13.3 and by the *GSTT1* gene located on chromosome 22q11.2, respectively [18,19,20]. The presence of the homozygous null polymorphisms results in the total absence of the enzymes’ activity; consequently, in estrogen metabolism, it will compromise the detoxification of catechol estrogens, known to contribute to hormone-induced carcinogenesis through DNA adducts formation [3,19,20].

As previously referred, mutations acquisition during a lifetime, particularly in low penetrance genes, are a risk factor for breast cancer and probably have a higher impact in breast cancer carcinogenesis in older women.

The polymorphism Val432Leu (*CYP1B1*) increases CYP1B1 activity, contributing to higher levels of catechol estrogens that are detoxified by Phase II enzymes. However, C677T polymorphism of *MTHFR* possibly affects 4-OH-E2 catalyzation by COMT; in addition, the total absence of GSTM1 and GSTT1, due to the null polymorphisms, will highly compromise the detoxification of catechol estrogens. Thus, we suppose that mutations acquisition during a lifetime might be related to breast cancer development at later ages.

In this regard, the present study was designed to investigate the impact of mutations acquisition in low penetrance genes during the lifetime in breast cancer development—more specifically, the impact of the null polymorphisms in *GSTM1* and *GSTT1* in breast cancer development at later ages.

## 2. Results

In the present study, 157 patients diagnosed with breast cancer were included, with a mean age of 63.71 years, as shown in Table 1. The majority of the patients were more than 50 years old (80.25%), and the mean ages for patients less than 50 years old was 43.65 years and 68.65 years for patients 50 years old or more.

Genotypes distribution of *GSTT1* and *GSTM1* according to breast cancer patient’s age are summarized in Table 2. The majority of breast cancer patients with the null genotype of *GSTT1* were 50 years old or more, and only 4 of the 47 cases identified with the null genotype were diagnosed with breast cancer before 50 years old (OR 3.497; 95% CI 1.149–10.641; *p*-value = 0.021). Concerning the correlation of *GSTM1* genotypes with the age of the patients, the majority of the cases 50 years old or more presented the null genotype; however, the distribution of present and null genotypes in patients less than 50 years old was similar (OR 1.973; 95% CI 0.892–4.363; *p*-value = 0.090).

A two-way combination of *GSTT1* and *GSTM1* genotypes with the age of the patients at breast cancer diagnosis was also performed (Table 3). The majority of those with the *GSTT1* null genotype and *GSTM1* present genotype were diagnosed at ages equal to or greater than 50 years old (OR 6.588; 95% CI 0.796–54.558; *p*-value = 0.050), and 29 of the 32 cases with both null genotypes were also identified in patients at later ages (OR 4.549; 95% CI 1.204–17.181; *p*-value = 0.018).

The null polymorphism of *GSTT1* was also combined with the polymorphism Val432Leu of *CYP1B1*. As represented in Table 4, the combination of the null genotype of *GSTT1* with the altered allele of *CYP1B1* were identified in 39 patients; of those, 35 were 50 years old or more at the diagnosis of breast cancer (OR 4.167; 95% CI 1.159–14.979; *p*-value = 0.022).

The combined analysis of *GSTM1* null polymorphism with Val432Leu of *CYP1B1* was also performed.

In Table 5, there were identified 47 patient carriers of the altered allele of *CYP1B1* with the genotype of *GSTM1* present; of those, 38 were 50 years old or more (OR 3.378; 95% CI 1.038–10.992; *p*-value = 0.038). The number of patients with null genotype of *GSTM1* and homozygous wild type genotype of *CYP1B1* diagnosed at later ages was 19 (OR 7.6; 95% CI 1.350–42.799; *p*-value = 0.013). Concerning patients with the presence of both polymorphisms, *GSTM1* null genotype and carriers of the altered allele of *CYP1B1*, 59 were 50 years old or more at diagnosis and only 12 were younger ages (OR 3.933; 95% CI 1.286–12.029; *p*-value = 0.012).

Additionally, the combination of *GSTT1* with the polymorphism C677T of *MTHFR* was performed, and results are summarized in Table 6. Among the 32 patients with both null genotype of *GSTT1* and altered allele of *MTHFR*, only 2 patients were diagnosed with breast cancer before 50 years old (OR 5; 95% CI 1.009–24.773; *p*-value = 0.034).

All these results indicate that the cumulative presence of polymorphisms related to catechol estrogens metabolism might be related with breast cancer diagnosis at later ages.

## 3. Discussion

Breast cancer has long been associated with estrogens exposure, lifestyle and genetic conditions [3,4,8]. Besides the inherited genetic alterations, acquired polymorphisms and genomic instability during life might predispose women to breast cancer [21]. High levels of estrogen have been associated with breast cancer risk, contributing to cellular proliferation of mutated cells and eventually increasing the opportunity for new mutations, leading to tumor progression [22]. In this regard, in breast cancer the estrogen metabolic pathway is of main importance due to estrogens detoxification [23]. Lifetime estrogen exposure and alterations in the enzymes involved in estrogens detoxification might influence cellular hormone-dependent growth [23,24]. Thus, due to mutations in genes that codify enzymes of the estrogen metabolic pathway, it is pertinent to identify subgroups of individuals more susceptible to the exposure of high levels of estrogens [10]. We performed an evaluation of the age at diagnosis of hormone-dependent breast cancer associated with mutations that might compromise the estrogen metabolic pathway.

Genetic mutations in GSTs, mainly the null genotype of *GSTT1* and *GSTM1* are considered a risk factor for breast cancer [16,17], but little is known about the presence of these polymorphisms and age at the diagnosis of breast cancer.

The age at breast cancer diagnosis was correlated with the null polymorphism of *GSTM1* and *GSTT1*, both alone or in association. As well, age at breast cancer diagnosis was correlated with *GSTM1* and *GSTT1* genotypes, together with other polymorphisms in the estrogen metabolic pathway, namely with *CYP1B1* Val432Leu and *MTHFR* C677T polymorphisms. The genotyping of these polymorphisms was performed in 157 women with histologically confirmed hormone-dependent breast cancer from Hospital Centre of Cova da Beira.

GSTM1 and GSTT1 are phase II enzymes that detoxify catechol estrogen quinones through the conjugation of GSH [3]. The absence of these enzymes, due to the null polymorphism of *GSTM1* and *GSTT1*, compromises the detoxification and allows the accumulation of catechol estrogens, leading to DNA adducts formation [17]. In the present study, we verified that the majority of breast cancer patients with the null genotype of *GSTT1* were 50 years old or more (*p*-value = 0.021), and in a two-way association of *GSTT1* and *GSTM1* genotypes with age, we verified similar results: the majority of the elderly patients had both polymorphisms (*p*-value = 0.018). We suppose that prolonged exposure to estrogen levels combined with an inefficient detoxification due to *GSTM1* and *GSTT1* null genotype are related to breast cancer development at later ages. This fact can be explained by the accumulation of catechol estrogens and DNA adducts formation during a lifetime, which culminate in breast cancer development.

Furthermore, there is the cumulative factor of other polymorphisms in the metabolic pathway of estrogens. It was verified that women over 50 years old with *GSTM1* null polymorphism are also carriers of the Val allele of *CYP1B1* Val432Leu polymorphism (*p*-value = 0.012), and similar results were found for the presence of *GSTT1* null polymorphism and Val allele of *CYP1B1* (*p*-value = 0.022). The *CYP1B1* Val432 allele promotes higher activity of CYP1B1, leading to higher levels of 4-OH-E2 and a consequent increase of carcinogenic catechol estrogen quinones [3,10,23]. The absence of *GSTM1* and *GSTT1* compromise the detoxification of these high levels of catechol estrogens, which eventually will contribute to tumor development in later ages.

The polymorphism of *MTHFR* C677T promotes a lower activity of MTHFR and a consequent decrease of detoxification via COMT. A two-way association of *MTHFR* C677T and *GSTT1* null genotype was performed and we verified that the majority of women carriers of both altered T allele of *MTHFR* C677T and *GSTT1* null genotype were 50 years old or more at the age of diagnosis (*p*-value = 0.034). These results might be explained by the fact that the metabolic pathway is extremely compromised due to inexistent GSTT1 and low COMT activity; low levels of Phase II enzymes highly compromise 4-OH-E2 detoxification and eventually will contribute to tumor development due to inefficient estrogens detoxification during reproductive life.

The four low penetrating genes analyzed in the present study indicate that mutations in enzymes that lead to an inefficient detoxification associated with exposure to endogenous or exogenous estrogens during life, might be a trigger to hormone-dependent breast cancer development at later ages. Once estrogens exert their biological activity by binding to their receptors, the referred inefficient detoxification might predispose women to the development of estrogen receptor positive (ER+) breast cancer.

These results are not only pertinent to understanding the influence polymorphisms in the metabolic pathway of estrogens but are also of main importance when considering hormone replacement therapy (HRT). Women with these genotypes are at higher risk of developing breast cancer; continuing the exposure to estrogens through therapy might increase the risk, and once these polymorphisms lead to inefficient estrogens detoxification, these estrogens might turn biologically active by binding to ER, contributing to the development of hormone-dependent breast cancer. This study indicates that it would be pertinent to evaluate in clinical practice the genotypes of each woman when considering HRT.

Once it was expected that by 2035 the number of new cancer cases would double among the older population (65 years old or more) [25]; it is important to identify the mechanisms that might contribute to this tendency. In the particular case of breast cancer development in older women, this study indicates that SNPs in low penetrance genes might have a profound impact on tumor development. Thus, genetic evaluation will contribute to identifying women at higher risk of breast cancer development at later ages and also women who are candidates for HRT. If preventive measures are taken, in the future, it will eventually be possible to forestall the expected increase of breast cancer cases in older women.

## 4. Materials and Methods

### 4.1. Study Population

The study group consisted of a total of 157 women with histologically confirmed hormone-dependent (ER positive) breast cancer diagnosed at Child and Women Health Department, Gynaecologic Oncology Division of Hospital Centre of Cova da Beira, Covilhã, Portugal. Informed consent was obtained from all individual participants included in the study. The study was approved by the Institutional Review Board of Hospital Centre of Cova da Beira, Covilhã, Portugal.

### 4.2. DNA Extraction

Blood was collected by venous puncture to EDTA tubes and genomic DNA was isolated using Wizard Genomic DNA purification kit (Promega) according to the instructions of the manufacturer and stored at 4 °C.

### 4.3. Genotyping

Genotyping of *GSTM1* and *GSTT1* (present or null polymorphism) was performed by multiplex polymerase chain reaction (PCR) with the co-amplification of β-globin gene as positive control, as previously described by our group [16].

Regarding to *CYP1B1* polymorphism and *MTHFR* polymorphism, the genotyping was performed by PCR-restriction fragment length polymorphism (RFLP). For both *CYP1B1* Val432Leu and *MTHFR* C677T polymorphisms, the amplification of the fragments containing the polymorphism in study was carried out in a total volume of 50 μL, and contained 10 pmol of each primer, 1.5 mM of MgCl_2_, 100 nM of each deoxynucleotide triphosphate, 1 unit of DreamTaq DNA polymerase and 100 ng of genomic DNA, using a MyCycler thermal cycler (Bio-Rad).

#### 4.3.1. CYP1B1

The genotyping of *CYP1B1* Val432Leu was performed with slight changes to the protocol of Zheng et al. [9].

The primers set used for *CYP1B1* Val432Leu genotyping were:

Forward primer: 5′-TCACTTGCTTTTCTCTCTCC-3′

Reverse primer: 5′-AATTTCAGCTTGCCTCTTG-3′.

The reaction mixtures were pre-incubated for 1 min at 94 °C. PCR conditions were 30 s at 94 °C, 30 s at 60 °C and 45 s at 72 °C for 35 cycles. The final extension was at 72 °C for 7 min. The amplified DNA fragment had 650bp. The PCR product was digested by Eco57I (Fermentas, St. Leon-Rot, Germany) restriction endonuclease for 16 h. Digested fragments were electrophoresed through 3% agarose gels stained with GreenSafe Premium (NZYTech, Lisbon, Portugal).Genotypes were distinguished by the pattern of fragments created by the digestion. Homozygous wild type Val/Val genotype was identified by the non-digested fragment of 650 bp; homozygous Leu/Leu genotype was identified by two digested fragments of 340 bp and 310 bp; heterozygous Val/Leu genotype was defined by presence of all fragments 650 bp, 340 bp and 310 bp.

#### 4.3.2. MTHFR

The genotyping of *MTHFR* C677T was performed with slight changes to the protocol of Reljic et al. [26].

The primers set used for *MTHFR* C677T genotyping were:

Forward: 5′- TGAAGGAGAAGGTGTCTGGGGGA-3′

Reverse: 5′- AGGACGGTGCGGTGAGAGTG-3′.

The PCR conditions were 30 s at 94 °C, 30 s at 61 °C and 1 min at 72 °C, for 30 cycles. The final extension was at 72 °C for 2 min. The amplified DNA fragment had 198 bp. The PCR product was digested by 1 U of HinfI (Fermentas, St. Leon-Rot, Germany) restriction endonuclease for 16 h. Digested fragments were electrophoresed through 3% agarose gels stained with GreenSafe Premium (NZYTech, Lisbon, Portugal). Homozygous wild type CC genotype was identified by the non-digested fragment of 198 bp; homozygous TT genotype was identified by two digested fragments of 175 bp and 23 bp; heterozygous CT genotype was defined by presence of all fragments 198 bp, 175 bp and 23 bp.

### 4.4. Statistical Analysis

In order to examine the association between genotypes in women with breast cancer, statistical analysis was performed using SPSS, version 23. Chi-squared tests were used, considering a statistical significance when *p*-value was <0.05.

## Figures and Tables

**Table 1:** Age characteristics of the 157 breast cancer patients included in the study.

	Patients *n* (%)	Mean (SD, Min–Max)
Total	157	
Age		63.71 (13.777, min 34–max 95)
<50	31 (19.75%)	43.65 (4.270, min 34–max 49)
≥50	126 (80.25%)	68.65 (10.395, min 50–max 95)

**Table 2:** Association of *GSTT1* and *GSTM1* genotypes with breast cancer patients’ age.

Genotype	Age, *n* (%)		OR (95% CI) ^a^	*p*-Value
	<50	≥50		
*GSTT1*				
Present	27 (17.2)	83 (52.9)		1
Null	4 (2.5)	43 (27.4)	3.497 (1.149–10.641)	0.021 *
*GSTM1*				
Present	17 (10.8)	48 (30.6)		1
Null	14 (8.9)	78 (49.7)	1.973 (0.892–4.363)	0.090

^a^ OR, odds ratio; CI, confidence interval; *, indicates a significant result.

**Table 3:** Association between *GSTT1* and *GSTM1* genotypes combination and patient age at diagnosis of breast cancer.

*GSTT1*	*GSTM1*	Age, *n* (%)		OR (95% CI) ^a^	*p*-Value
		<50	≥50		
Present	Present	16 (10.2)	34 (21.7)		1
Present	Null	11 (7)	49 (31.2)	2.096 (0.866–5.072)	0.097
Null	Present	1 (0.6)	14 (8.9)	6.588 (0.796–54.558)	0.050 *
Null	Null	3 (1.9)	29 (18.5)	4.549 (1.204–17.181)	0.018 *

^a^ OR, odds ratio; CI, confidence interval; *, indicates a significant result.

**Table 4:** Association between *GSTT1* and *CYP1B1* Val432Leu genotypes combination and patient age at diagnosis of breast cancer.

*GSTT1*	*CYP1B1* Val432Leu	Age, *n* (%)		OR (95% CI) ^a^	*p*-Value
		<50	≥50		
Present	Leu/Leu (WT)	10 (6.4)	21 (13.4)		1
Present	Leu/Val + Val/Val	17 (10.8)	62 (39.5)	1.737 (0.689–4.378)	0.239
Null	Leu/Leu (WT)	0	8 (5.1)	NA ^b^	0.062
Null	Leu/Val + Val/Val	4 (2.5)	35 (22.3)	4.167 (1.159–14.979)	0.022 *

^a^ OR, odds ratio; CI, confidence interval; ^b^ Not applicable; *, indicates a significant result.

**Table 5:** Association between *GSTM1* and *CYP1B1* Val432Leu genotypes combination and patient age at diagnosis of breast cancer.

*GSTM1*	*CYP1B1* Val432Leu	Age, *n* (%)		OR (95% CI) ^a^	*p*-Value
		<50	≥50		
Present	Leu/Leu (WT)	8 (5.1)	10 (6.4)		1
Present	Leu/Val + Val/Val	9 (5.7)	38 (24.2)	3.378 (1.038–10.992)	0.038 *
Null	Leu/Leu (WT)	2 (1.3)	19 (12.1)	7.600 (1.350–42.799)	0.013 *
Null	Leu/Val + Val/Val	12 (7.6)	59 (37.6)	3.933 (1.286–12.029)	0.012 *

^a^ OR, odds ratio; CI, confidence interval; *, indicates a significant result.

**Table 6:** Association between *GSTT1* and *MTHFR* C677T genotypes combination and patient age at diagnosis of breast cancer.

*GSTT1*	*MTHFR* C677T	Age, *n* (%)		OR (95% CI) ^a^	*p*-Value
		<50	≥50		
Present	CC (WT)	10 (6.4)	30 (19.1)		1
Present	CT+TT	17 (10.8)	53 (33.8)	1.039 (0.422–2.557)	0.933
Null	CC (WT)	2 (1.3)	13 (8.3)	2.167 (0.415–11.302)	0.351
Null	CT+TT	2 (1.3)	30 (19.1)	5 (1.009–24.773)	0.034 *

^a^ OR, odds ratio; CI, confidence interval; *, indicates a significant result.

## Data Availability

The data presented in this study are available within the article or on request from the corresponding author.
